# Prompt Diagnosis of Ethylene Glycol Intoxication by an Unusual “Lactate Gap”: A Case Report

**DOI:** 10.5811/cpcem.2021.12.54928

**Published:** 2022-01-28

**Authors:** Jie Tang

**Affiliations:** Alpert Medical School of Brown University, Division of Kidney Diseases and Hypertension, Providence, Rhode Island

**Keywords:** case report, ethylene glycol intoxication, lactate

## Abstract

**Introduction:**

Ethylene glycol intoxication can be lethal if diagnosis is delayed. Often, prompt diagnosis may need to be based on indirect laboratory findings.

**Case report:**

We present a case of severe ethylene glycol intoxication whose diagnosis was based on an unusual “lactate gap.” The patient responded well to the treatment and had a full recovery.

**Conclusion:**

A “lactate gap” can be helpful in establishing a diagnosis of ethylene glycol intoxication.

## INTRODUCTION

Ethylene glycol (EG) intoxication can lead to severe metabolic acidosis and acute kidney injury and can be lethal if diagnosis is delayed. Direct measurement of ethylene glycol is often not readily available. Prompt recognition and intervention may require recognition of indirect laboratory findings. Here, the author presents a case of EG intoxication whose diagnosis was based on an unusual “lactate gap.”

## CASE REPORT

A 61-year-old male was found unresponsive at home by police. He reportedly texted his friend stating that he was going to “hurt himself,” which prompted his friend to call the police. His past medical history was significant for human immunodeficiency virus infection with history of Kaposi sarcoma (recent viral load undetectable), autism spectrum disorder, depression, and post-traumatic stress disorder. Home medications included atorvastatin 10 milligrams (mg) daily and Genvoya (elvitegravir, cobicistat, emtricitabine, tenofovir alafenamide) 150/150/200/10 mg daily. He lived alone, smoked one-quarter pack of cigarettes a day, and had a history of heavy alcohol use in the past. Family history was noncontributory.

On presentation, he was afebrile, heart rate was 60 beats per minute, blood pressure was 134/71 millimeters of mercury (mm Hg), respiratory rate was 16 breaths per minute, and oxygen saturation was 100% on room air. However, he was unresponsive with a Glasgow Coma Scale score of three. The rest of the physical exams was unrevealing. Laboratory testing showed acute kidney injury with a high anion gap metabolic acidosis. We also observed a large discrepancy in lactate measurements between the whole blood and serum samples (Table).

His liver function test and complete blood count with differential were unremarkable. Blood salicylate and ethanol levels were not detected, and urine drug screen was negative. Electrocardiogram, head computed tomography and chest radiograph were unremarkable. He was oliguric at the time and was intubated for airway protection.

Based on his clinical presentation and the discrepancy between his whole blood and serum lactate measurements, an EG ingestion was suspected. Urine sediment showed calcium oxalate monohydrate crystals (Image). Serum osmolality was measured subsequently and showed an osmolar gap of 56. Fomepizole was started with an initial loading dose of 15 mg/kilogram (kg) followed by an urgent four-hour hemodialysis (HD) session 3.5 hours later. Subsequent fomepizole dosing frequency at the standard 10 milligrams (mg) per kg (mg/kg) dose were adjusted according to his dialysis schedule. His blood EG level after the initial HD session was 127 mg per deciliter (mg/dL).

Considering the clearance of EG via standard HD is about 150 milliliters per minute,^1^ a three-hour session in a 100-kg person will clear 45% of EG. Thus, an extra three-hour HD session brought the patient’s EG level down to 71 mg/dL as predicted. He received another four-hour HD session the next day, and his EG level went down to 31 mg/dL. His whole blood lactate level was also normalized soon after. He subsequently recovered his kidney function, was extubated, and discharged to the in-patient psychiatric service.

**CPC-EM Capsule**

What do we already know about this clinical entity?

*In cases of ethylene glycol (EG) intoxication, direct measurement of ethylene glycol may not be available. Recognition may have to rely on indirect lab findings*.

What makes this presentation of disease reportable?

*Timely diagnosis of EG intoxication was made based on a “lactate gap.” which can be quickly obtained using a point-of-care analyzer*.

What is the major learning point?

*Ethylene glycol intoxication can lead to a “lactate gap,” which is the difference in lactate measured using two different analyzers*.

How might this improve emergency medicine practice?

*The “lactate gap” can be a surrogate marker for the ethylene glycol metabolite, leading to early diagnosis and initiation of effective treatment*.

## DISCUSSION

Ethylene glycol is metabolized via alcohol dehydrogenase, aldehyde dehydrogenase, and lactate dehydrogenase (LDH). While oxalate is the metabolite primarily responsible for end-organ damage including kidney injury, glycolic acid is mostly responsible for anion gap acidosis. Clinical manifestations include altered metal status (due to the parent compound), and organ damage (from oxalate deposition). The presenting signs and symptoms are often non-specific, with signs of initial central nervous system depression occurring within 12 hours after ingestion. Cardiopulmonary manifestations develop approximately 24 hours after the ingestion and are characterized by hyperventilation, tachycardia, and hypertension. Ethylene glycol poisoning requires rapid recognition and early treatment in a time-dependent fashion as it can lead to permanent organ damage and high mortality.^2^,^3^ However, its diagnosis is challenging due to lack of ingestion history and a lack of readily available assays for the toxic alcohols.

Despite these diagnostic obstacles, certain laboratory clues can help clinicians identify the causative toxic agent. The presence of metabolic acidosis with high anion gap and osmolar gap should raise suspicion for toxic alcohol ingestions. The presence of calcium oxalate crystal in urine sediment is an important clue for EG intoxication. The “lactate gap” is the difference in values obtained from two different analyzer methods to detect a falsely elevated lactate level. The radiometry method for lactate is commonly used in point-of-care testing reported in the blood gas. It uses the enzyme L-lactate oxidase to accelerate the oxygenation of L-lactate producing hydrogen peroxide and pyruvate. The L-lactate concentration is then computed from the measured hydrogen peroxide concentration. Glycolic acid, a metabolite of EG, cross-reacts with L-lactate oxidase and produces a significant amount of hydrogen peroxide leading to a falsely elevated lactate level. Serum lactate levels, however, are typically computed using a non-radiometry method. Analyzers, such as the iSTAT, Bayer, or Beckman and Vitros, measure LDH (instead of lactate oxidase) activity, which is not affected by glycolic acid. In our case, the “lactate gap” served as a surrogate marker for the EG metabolite that prompted the clinician to an early diagnosis and initiation of effective treatment.

## CONCLUSION

Timely diagnosis of toxic alcohol ingestion is essential to improve clinical outcome. However, the detection of parent alcohol or its metabolites in blood is labor intensive and time consuming. In suspected cases of ethylene glycol ingestion, “Lactate gap” can be a quick and helpful laboratory clue for ethylene glycol intoxication.

## Figures and Tables

**Image:**
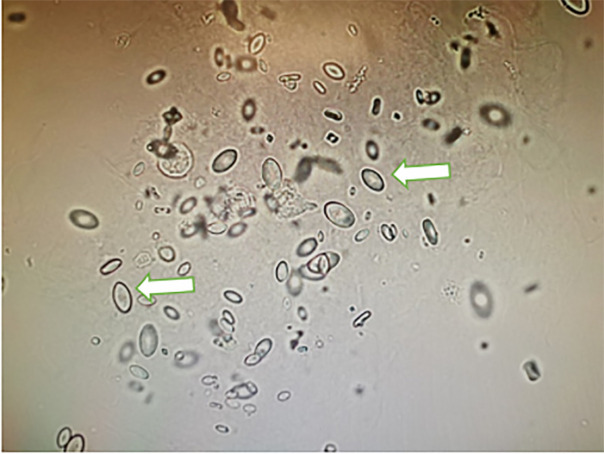
Urine sediment examination showed characteristic calcium oxalate monohydrate crystals (white arrows).

**Table:** Laboratory values in patient who presented with altered mental status.

Measurement (units)	Reference value	2 months prior	On admission
Blood urea nitrogen (mg/dL)	7–20		20
Serum creatinine (mg/dl)	0.6–1.2	1.1	1.7
Serum bicarbonate (mEq/L)	22–32		12
Anion gap	3–13		25
Serum lactate (mEq/L)	0.2–1.9		0.5
Venous blood gas pH	7.32–7.42		7.10
Venous blood gas partial pressure of carbon dioxide (pCO_2_) (mm Hg)	42–50		41
Whole blood lactate (mEq/L)	0.2–1.9		17

*mg/dL*, milligrams per deciliter; *mEq/L*, milliequivalents per liter; *pCO_2_*, partial pressure of carbon dioxide; *mm Hg*, millimeters of mercury.
